# Single-cell multi-omics sequencing and its applications in studying the nervous system

**DOI:** 10.52601/bpr.2021.210031

**Published:** 2022-06-30

**Authors:** Chaoyang Wang, Xiaoying Fan

**Affiliations:** 1 Bioland Laboratory (Guangzhou Regenerative Medicine and Health Guangdong Laboratory), Guangzhou 510005, China; 2 The Fifth Affiliated Hospital of Guangzhou Medical University, Guangzhou 510700, China

**Keywords:** Single-cell sequencing, Multi-omics, Cell heterogeneity, Nervous system

## Abstract

Single-cell sequencing has become one of the most powerful and popular techniques in dissecting molecular heterogeneity and modeling the cellular architecture of a biological system. During the past twenty years, the throughput of single-cell sequencing has increased from hundreds of cells to over tens of thousands of cells in parallel. Moreover, this technology has been developed from sequencing transcriptome to measure different omics such as DNA methylome, chromatin accessibility, and so on. Currently, multi-omics which can analyze different omics in the same cell is rapidly advancing. This work advances the study of many biosystems, including the nervous system. Here, we review current single-cell multi-omics sequencing techniques and describe how they improve our understanding of the nervous system. Finally, we discuss the open scientific questions in neural research that may be answered through further improvement of single-cell multi-omics sequencing technology.

## INTRODUCTION

Single-cell sequencing was first reported in 2009, following which this technology has rapidly developed and has been adopted across various research areas, including the nervous system (Mu *et al.* 2019; Tang *et al.* 2009). The nervous system is one of the most essential biological systems in higher animals, composed of large numbers of cell types of different physiological functions. Cells are the fundamental structural units of metazoans. Thus, clarifying different cell types, their molecular signatures, and the relationships between them are necessary steps to elucidate the complexity of the nervous system.

The use of single-cell sequencing technology to investigate cell types in the nervous system was first reported in 2014, in which 301 cells from the human cortex germinal zone were sequenced. These cells could be designated as different progenitors and neurons according to their expression of classical marker genes (Pollen *et al.* 2014). According to single-cell RNA-seq data, FGF, Wnt, and Notch signal pathways were confirmed to play important roles in neurogenesis (Basak *et al.* 2012; Bengoa-Vergniory and Kypta 2015; Engler *et al.* 2018; Hatakeyama *et al.* 2004; Krol *et al.* 2011; Sahara and O'Leary 2009). These findings demonstrated the utility of the single-cell technique as a tool for investigating neural development. However, this original study drew conclusions with a very limited cell population. With further technical development, researchers can accomplish sequencing from low-throughput to as many as tens of thousands of single cells for one sample (Cardona-Alberich *et al.* 2021; Dulken *et al.* 2019; Hrvatin *et al.* 2018; Hu *et al.* 2017; Mu *et al.* 2019; Tasic *et al.* 2018; Vanlandewijck *et al.* 2018). One recent project even obtained single-cell data from over two million cells (Cao *et al.* 2019). These studies provide detailed information on the nervous system at the single-cell level, dissecting various cell types and clarifying the complex construction of the brain.

So far, the majority of single-cell sequencing studies have focused on the transcriptome, as the transcriptome reflects both partial genomic sequence information and partial protein translation information via its role in the central dogma, produced by transcription using genomic DNA as a template and a template itself in translating the proteome. In fact, proteins are the executors of cell function, and mRNA levels can roughly infer the types and levels of proteins. On the other hand, protein production is not merely determined by the abundance of the corresponding mRNA. Protein quantity is also affected by other factors including RNA modifications and regulators such as non-coding RNA. Therefore, the transcriptome only reveals partial information on the state of the cell. Further, gene expression is affected by many epigenetic factors including genomic mutation, DNA methylation, histone modification, chromatin accessibility, and three-dimensional (3D) conformation. As the transcriptome is controlled by such a complex process, it is very unstable between different cells, even within a defined cell type.

Previous studies argue that patterns of DNA methylation and chromatin accessibility should be more accurate markers of cell type, whereas the transcriptome reflects the physiological state of cells (Kozlenkov *et al.* 2016; Luo *et al.* 2019; Mo *et al.* 2015; Zhu *et al.* 2020). Therefore, many different methods have been applied to detect different omics beyond the transcriptome at a single-cell resolution. For example, using bisulfite treatment to convert dCTP to dUTP, the DNA methylomes of more than 6,000 single nuclei from human and mouse frontal cortices were sequenced. There were 16 and 21 neuronal subtypes identified in the mouse and human cerebral cortex, respectively. The human brain was specifically identified with a subtype of cortical layer six excitatory neurons and an inhibitory neuron subtype (Luo *et al.* 2017). An additional study using high-throughput single-cell ATAC-seq (Assay for Targeting Accessible-Chromatin with high-throughput sequencing) showed that chromatin accessibility patterns could also distinguish neural subtypes in the human cerebral cortex and cerebellum, and the authors identified a pool of important *cis*-regulators for specific cell types (Lake *et al.* 2018).

Cellular heterogeneity is determined in multiple dimensions beyond the transcriptome. A single omics profile can help us to understand the physiological state of cells in one aspect, but bias also exists when measuring only one modality, preventing us from comprehensively resolving the heterogeneity of cells. Different omics as approaches can provide complementary information; the half-life of mRNA is short while covalent modifications of DNA, and some histone modifications, can persist for months to years (Armand *et al.* 2021; Chen *et al.* 2008; Colomé-Tatché and Theis 2018; Maze *et al.* 2015). As such, epigenetic factors are complementary to the transcriptome and can be used beyond gene expression levels to more accurately define cell types (Maze *et al.* 2015). In addition, different omics approaches reveal coordinated responses to signals from endogenous development and exogenous stimuli. Therefore, to better understand cellular heterogeneity and functions, single-cell multi-omics sequencing is gaining popularity, and is widely applied to investigate biological questions. In this review, we focus on the application of single-cell multi-omics sequencing in the nervous system. Existing reviews also summarize single-cell single-omics sequencing and its application to other research fields (Ma *et al.* 2020a; Mu *et al.* 2019; Zhu *et al.* 2019).

Currently there are several methods capable of measuring at least two omics layers in a single cell, most of which simultaneously measure the transcriptome and an additional omics layer, such as genomic mutation, DNA methylation, chromatin accessibility, *etc*. (Table 1). Below, we highlight seven categories of single-cell multi-omics approaches to discerning the structure of the nervous system. These methods are ordered according to the frequency of application to the nervous system and the timeline of method development.

**Table 1:** The reported multi-omics technologies

Method	Multi-omics	References
scMT-seq	5mC and RNA	Hu *et al*. 2016
scM&T-seq	5mC and RNA	Angermueller *et al*. 2016
scGEM	5mC and RNA	Cheow *et al*. 2016
snmCT	5mC and RNA	Luo *et al*. 2018
scTrio-seq	RNA, 5mC, and gDNA	Bian *et al*. 2018; Hou *et al*. 2016
scNOMe-seq	5mC and ChrA	Guo *et al*. 2017b; Pott 2017
scCOOL-seq	5mC, gDNA and ChrA	Guo *et al*. 2017a
iscCOOL-seq	5mC, gDNA, and ChrA	Gu *et al*. 2019
snmC2T	5mC, RNA and ChrA	Luo *et al*. 2019
scNOMeRe-seq	5mC, RNA and ChrA	Wang *et al*. 2021
scNMT-seq	5mC, RNA and ChrA	Clark *et al*. 2018
sciCAR-seq	RNA and ChrA	Cao *et al*. 2018
scCAT-seq	RNA and ChrA	Liu *et al*. 2019
ATAC-RNA-seq	RNA and ChrA	Reyes *et al*. 2019
Paired-seq	RNA and ChrA	Zhu *et al*. 2019
SNARE-seq	RNA and ChrA	Chen *et al*. 2019
SHARE-seq	RNA and ChrA	Ma *et al*. 2020b
10X Single Cell Multiome ATAC^+^ Gene Expression	RNA and ChrA	https://www.10xgenomics.com/products/single-cell-multiome-atac-plus-gene-expression
Methyl-HiC	5mC and 3DChr	Li *et al*. 2019
snm3C-seq	5mC and 3DChr	Lee *et al*. 2019
Paired-Tag	RNA and HisM	Zhu *et al*. 2021
CoTECH	RNA and HisM	Xiong *et al*. 2021
Slide-seq	RNA and Spa	Rodriques *et al*. 2019
Spatial transcriptomics	RNA and Spa	Stahl *et al*. 2016
HDST	RNA and Spa	Vickovic *et al*. 2019
MERFISH	RNA and Spa	Moffitt and Zhuang 2016
STAR-map	RNA and Spa	Wang *et al*. 2018
seqFISH+	RNA and Spa	Eng *et al*. 2019
CITE-seq	RNA and Pro	Stoeckius *et al*. 2017
REAP-seq	RNA and Pro	Peterson *et al*. 2017
PLAYR	RNA and Pro	Frei *et al*. 2016
PEA/STA	RNA and Pro	Genshaft *et al*. 2016
SPARC	RNA and Pro	Reimegard *et al*. 2021
RNA: transcriptome; 5mC: DNA methylome; ChrA: chromatin accessibility; 3DChr: chromatin 3D structure; Spa: cell spatial information; HisM: histone modification; Pro: proteome; gDNA: genome mutations		

## TRANSCRIPTOME AND DNA METHYLOME

**Figure 1:**
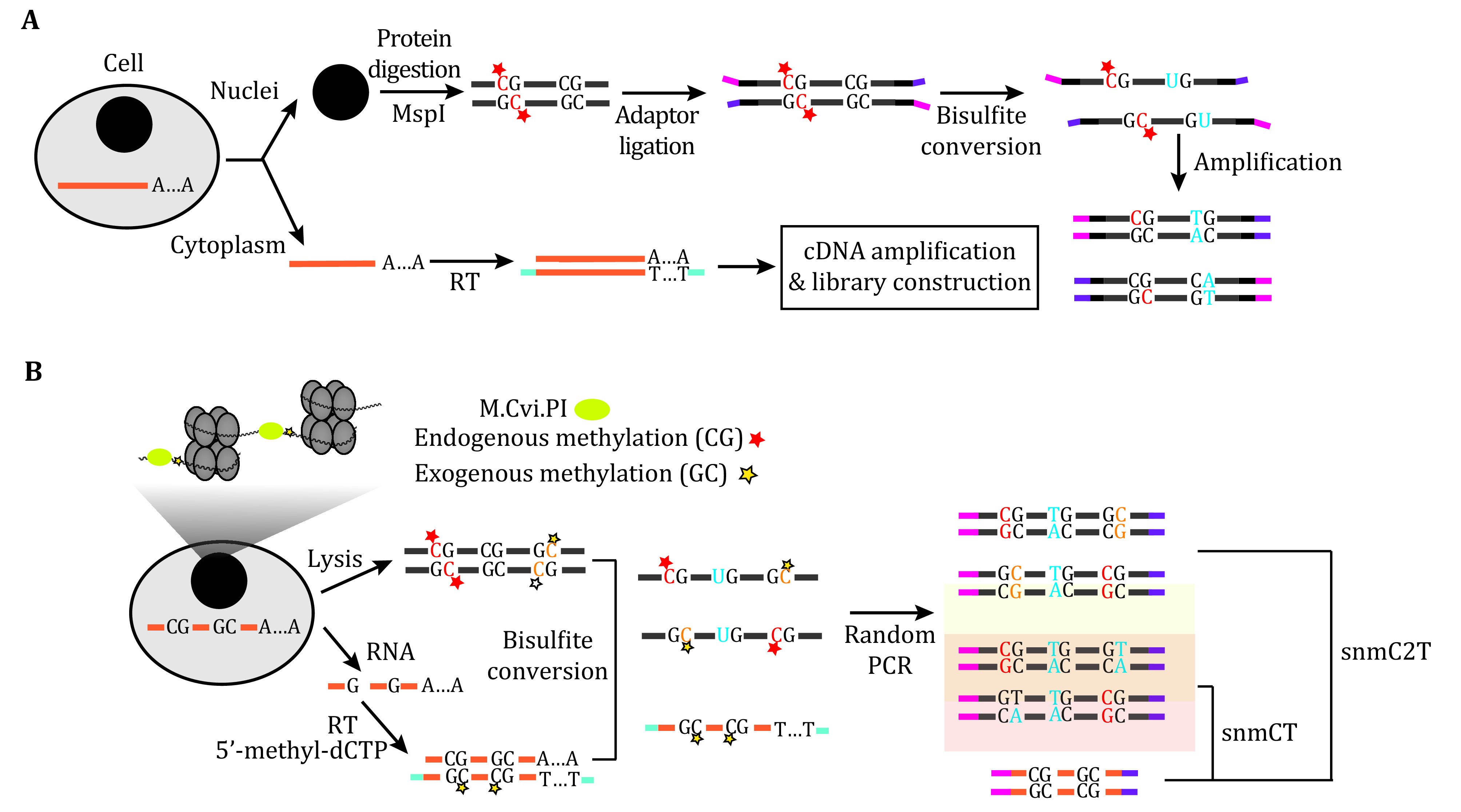
The schematic diagram of single-cell multi-omics sequencing methods detecting transcriptome, DNA methylation and chromatin accessibility. The schematic diagram of scM&T (**A**), snmCT and snmC2T (**B**). **A** In scM&T, genome and transcriptome of the same cell are originally obtained by separating the cytoplasm from the nuclei. Then single cell DNA methylome and transcriptome sequencing are carried out independently. **B** In snmCT, there is no step to physically separate transcriptome from the genome. Instead, the DNA and cDNA are amplified in the same reaction and distinguished according to the methylation of cytosine in the fragments. All reads from cDNA maintain cytosine while most C in genome fragments are converted into thymine. The snmC2T method further induces exogenous methylation (GC) to measure the chromatin accessibility level

Thus far, the transcriptome is the most informative dimension in single-cell sequencing. Regulation of the transcriptome by the DNA methylome has long been established (Hohn *et al.* 1996; Jones 2012; Schubeler 2015). Therefore, sequencing the transcriptome and the DNA methylome at a single-cell level can enhance cell type resolution and reveal gene expression regulatory networks (Armand *et al.* 2021). In this context, different methods had been developed such as scMT-seq, scM&T-seq, scGEM, snmCT and scTrio-seq (Angermueller *et al.* 2016; Bian *et al.* 2018; Cheow *et al.* 2016; Hou *et al.* 2016; Hu *et al.* 2016; Luo *et al.* 2018). The sensory neurons from adult mouse dorsal root ganglia were analyzed by scMT-seq (Hu *et al.* 2016). To obtain multi-omics data within the same cell, researchers manually separated the cytoplasm and nucleus to prepare libraries for the single-cell transcriptome and DNA methylome respectively (Fig. 1A). The direct correlation between mRNA expression and DNA methylation was confirmed in this study. For instance, the expression level of genes containing CpG island promoters showed a positive correlation with the methylation level of the corresponding gene body (Hu *et al.* 2016). In a similar way, mouse embryonic stem cells and hepatocellular carcinomas samples were analyzed and the researchers discovered regulatory pairs of methylated distal regulatory elements and downstream genes (Angermueller *et al.* 2016; Hou *et al.* 2016). For example, the expression of *Esrrb* negatively correlated with the DNA methylation level of several super enhancers (Angermueller *et al.* 2016; Papp and Plath 2012).

However, these technologies require physical separation of the cell nuclei and cytoplasm, leading to limited throughput and preventing wide application in the nervous system across complex cell types. High-throughput strategies have been used in other research fields. For example, scGEM was used in cancer studies (Cheow *et al.* 2016), and snmCT-seq was evaluated using H1 and HEK293 cells (Luo *et al.* 2018). These methods are suitable for nervous system exploration, especially considering snmCT-seq was further improved to also obtain chromatin accessibility information in the same cells, named snmC2T-seq (Luo *et al.* 2018, 2019). Based on analysis from snmC2T-seq, we infer that single-cell multi-omics with the transcriptome and DNA methylome could significantly enhance resolution for cell classification. The snmC2T-seq technology is discussed in a later section.

## TRANSCRIPTOME AND CHROMATIN ACCESSIBILITY

To initiate transcription, genomic DNA must be accessible for transcription factor binding. Chromatin accessibility is highly associated with gene expression regulation. In 2018, Lake *et al.* harvested different regions of the human cerebral cortex and cerebellum, and sequenced the single-cell transcriptome and chromatin accessibility separately. Their integrative analysis across both kinds of single-cell datasets identified a series of genes in which promoter/enhancer accessibility was highly correlated with expression level. Therefore, some key fate decision factors were identified with higher confidence (Lake *et al.* 2018). In the same way, the transcription factor OLIG2 was found to play a vital role in stem cell-derived oligodendrogenesis during injured spinal cord repair (Llorens-Bobadilla *et al.* 2020). However, separate sequencing on two sample sets could not reveal direct interactions between chromatin accessibility and gene expression.

**Figure 2:**
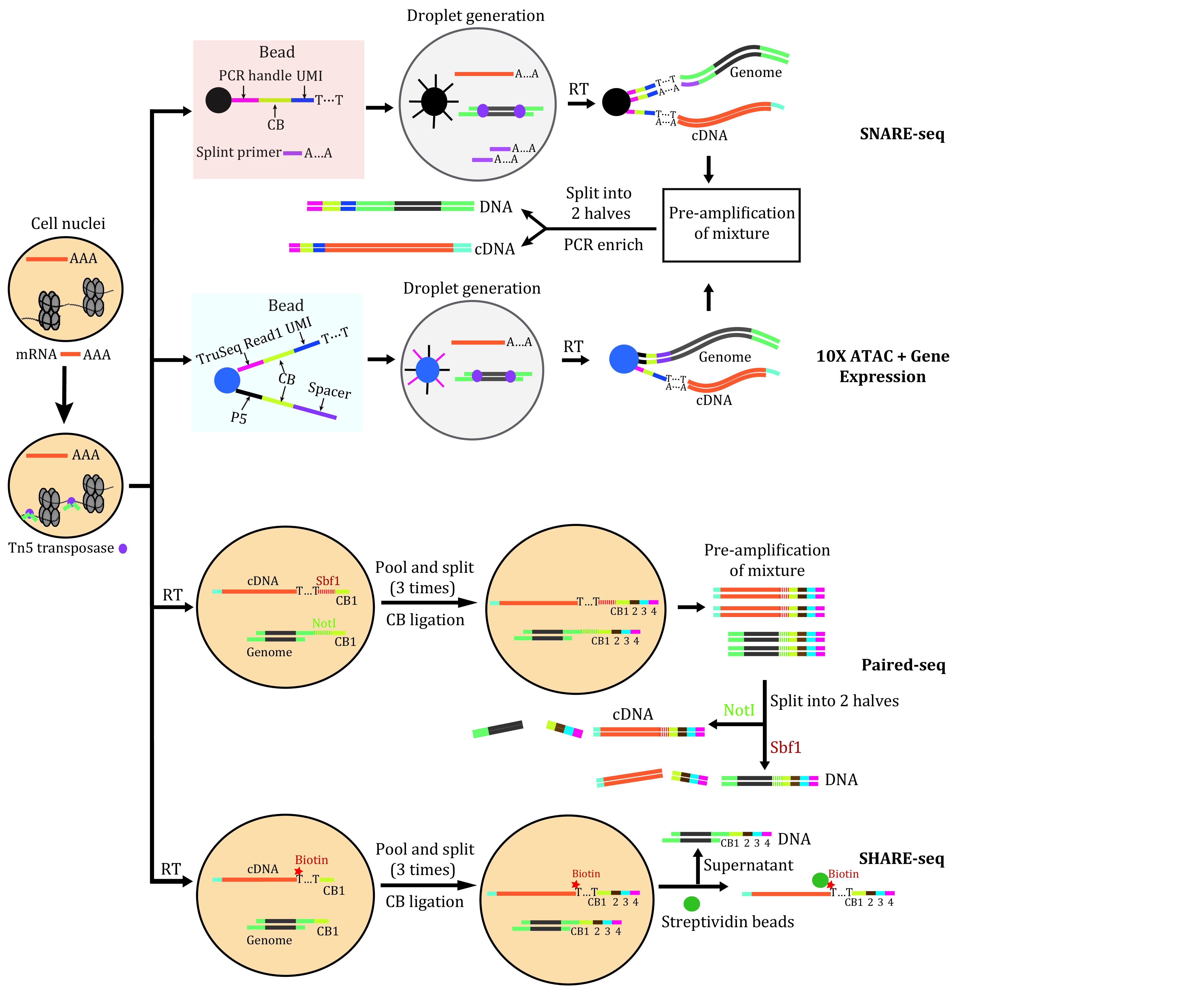
Schematic illustration of different methods simultaneously detecting transcriptome and chromatin accessibility. The four reported methods for high throughput sequencing of transcriptome and chromatin accessibility. SNARE-seq and 10X ATAC + Gene Expression kit are based on microfluidic systems, while Paired-seq and SHARE-seq use the pool-split barcoding strategy. They each adopts specific way for cDNA and genome enrichment. SNARE-seq uses a sprint primer to add cell barcode and UMI for the tagmentated DNA fragments; the gel beads of 10X ATAC + Gene Expression kit contain two different primers for RNA and tagmentated DNA capture, and these two methods enriched each modality by amplifying with different primers; Paired-seq and SHARE-seq use the same PCR primers for both cDNA and DNA fragments, while the former induces different endonuclease site to enrich each modality and the later uses biotin to separate cDNA and DNA fragments before amplification. CB: cell barcode; RT: reverse transcription

Different methods have been developed to achieve simultaneous detection of chromatin accessibility and gene expression in the same cells with high throughput in recent years, including sciCAR-seq, scCAT-seq, ATAC-RNA-seq, SNARE-seq, Paired-seq, and SHARE-seq (Table 1) (Cao *et al.* 2018; Chen *et al.* 2019; Liu *et al.* 2019; Ma *et al.* 2020b; Reyes *et al.* 2019; Zhu *et al.* 2019). The company 10X genomics has developed the commercial Next GEM Single Cell Multiome ATAC + Gene Expression kit in 2020. All methods depend on the Tn5 transposase to obtain accessible genome fragments first, following which the nuclear transcriptome and genome fragments are synchronously pre-amplified. SNARE-seq and 10X Next GEM Single Cell Multiome ATAC + Gene Expression kit are both based on the microdroplet platform while Paired-seq uses the strategy of splitting and pooling to obtain abundant single-cell data (Fig. 2). In 10X Next GEM Single Cell Multiome ATAC + Gene Expression kit, there are two types of primers, each specific for capturing mRNA and transposed DNA, respectively. In contrast, SNARE-seq uses the same primer to capture both mRNA and transposed DNA by incorporating a splint primer (Fig. 2). Next, the pre-amplified mixture of cDNA and transposed DNA fragments is subdivided into two parts and prepared in different libraries using specific primers (Fig. 2). In Paired-seq, the authors used different restriction enzymes targeting the pre-designed sites in each amplicon to digest either the cDNA or the transposed DNA fragments in a sequencing library. SHARE-seq shared a similar strategy with Paired-seq, and they used biotin-labeled oligos to separate the cDNA from genomic DNA (Fig. 2) (Ma *et al.* 2020b).

Combining the chromatin accessibility dataset with the transcriptome dataset, finer distinctions can be made to elucidate cellular heterogeneity. For example, intermediate progenitor cells were designated as a single cluster by transcriptome, while the same population of cells was divided into three subclusters by SNARE-seq (Chen *et al.* 2019). Meanwhile, the cell-type boundaries were relatively unclear and some minor cell types were not detected when only referred by the single-cell ATAC-seq dataset (Chen *et al.* 2019). With single-cell pair-wise datasets, the direct correlation of gene expression with the accessibility of *cis*-regulatory elements was confirmed for cell type specific genes (Chen *et al.* 2019; Trevino *et al.* 2021; Zhu *et al.* 2019). By identifying potential regulatory programs during neurogenesis and pathogenesis, we can evaluate the risks of neural disorder diseases at much earlier stages. Trevino *et al.* used the 10X Next GEM Single Cell Multiome ATAC + Gene Expression kit to analyze the human fetal cerebral cortex, and they found that the chromatin states of lineage-defining transcription factors are already accessible before the progenitor cells enter the cell cycle (Trevino *et al.* 2021). The researchers also infer the regulatory impact of autism spectrum disorder (ASD) relevant non-coding genetic variants in different cell types based on the chromatin accessibility patterns, including identification of loss of function of NFIA during the pathogenesis of ASD (Iossifov *et al.* 2014). Specifically, one G to A mutation in the enhancer, located within the intron of NFIA, may disrupt accessibility and silence NFIA in some excitatory neurons (Trevino *et al.* 2021). In addition to marker genes, the ATAC-seq peaks can also act as biomarkers for specific cell clusters. In SHARE-seq, the authors also identified temporal sequential activation of genome regions and gene expression in mouse skin development. Therefore, the chromatin accessibility of enhancers has the potential to foreshadow gene expression and predict lineage choice prior to the expression of key genes (Ma *et al.* 2020b).

## TRANSCRIPTOME, DNA METHYLOME AND CHROMATIN ACCESSIBILITY

As mentioned above, both chromatin accessibility and DNA methylation are related to gene expression. Moreover, they can be analyzed at the single cell level by using different methods such as scNOMe-seq, scCOOL-seq and iscCOOL-seq (Gu *et al.* 2019; Guo *et al.* 2017a, b; Pott 2017). In order to understand cell state and the regulatory network more comprehensively, triple-omics sequencing including the transcriptome, DNA methylome, and chromatin accessibility within the same cell was developed. The reported methods include scNMT-seq, snmC2T-seq, and scNOMeRe-seq (Clark *et al.* 2018; Luo *et al.* 2019; Wang *et al.* 2021). All of these technologies adopted the same strategy by inducing exogenous GpC methylation to measure the open chromatin sites, while endogenous CpG methylation represents the DNA methylome. In scNMT-seq and scNOMeRe-seq, genomic DNA and mRNA in a single cell were physically separated. As such, these methods are not practicable for high throughput analysis, nor have these methods been applied to nervous system research yet. We instead focus on snmC2T-seq as an example, which is compatible with high-throughput operations such as split-pooling and the drop-seq platform.

In snmC2T-seq, the GpC methyltransferase M.CviPI was used to add a methyl group to accessible GpC sites. Furthermore, the researchers utilized 5’-methyl-dCTP instead of dCTP during cDNA synthesis to distinguish from genomic DNA after bisulfite conversion (Fig. 1B) (Luo *et al.* 2018, 2019). Using snmC2T-seq to combine the transcriptome with the DNA methylome and chromatin accessibility enabled investigation of fine-grained cell subtypes in the human brain with higher sensitivity. For instance, analysis relying on only gene expression pinpointed the genetic risk of schizophrenia across broad cortical neuronal cell populations, such as the neocortical somatosensory pyramidal cells and cortical interneurons (Skene *et al.* 2018). Triple-omics analysis accurately targeted intratelencephalic neuron types, including L1-3 CUX2, L4-5 FOXP2 and L5-6 PDZEN4 and interneuron subtype MGE CALB1 (Luo *et al.* 2019). This indicates that multi-omics has the ability to pinpoint more accurate cell populations that are responsible for the pathogenesis of psychiatric disorders.

Vestigial enhancers are usually hypomethylated elements that exhibit activity during embryonic development but lack activity in adult tissues. They can be good biomarkers for the genetic risk of neuropsychiatric traits, as the low methylation level is maintained constitutively (Hon *et al.* 2013). To find the cell types possibly responsible for the pathogenetic process, the researchers used a partitioned heritability analysis to investigate those vestigial enhancers (Luo *et al.* 2019). Combining the DNA methylome and chromatin accessibility with the genetic risk locus and genes identified by a genome-wide association study (GWAS), some vestigial enhancers in or around the genetic risk locus of certain psychiatric diseases were enriched in specific cells. For example, vestigial enhancers that had been reported to be responsible for ASD genetic risk also showed strong enrichment in L4 PLCH1 and L6 TLE4 cell populations, indicating that they may be the cell populations essential in ASD pathogenesis (Grove *et al.* 2019; Luo *et al.* 2019).

By using scNMT-seq, the negative correlation between promoter DNA methylation and chromatin accessibility in single mouse embryonic stem cells was confirmed (Clark *et al.* 2018; Thurman *et al.* 2012). According to correlation analyses of different omics layers, although DNA methylation showed dynamic correlations with gene expression, it was more widely correlated with gene expression than chromatin accessibility (Clark *et al.* 2018). Beyond the two regulators, other epigenetic layers also play essential roles in gene expression regulation, such as histone modification which was reported to be essential for the function of vestigial enhancers (Hon *et al.* 2013).

## TRANSCRIPTOME AND HISTONE MODIFICATIONS

Histone modifications varied greatly in their cellular specificity and relationship with cell-type-specific gene expression (Stadhouders *et al.* 2019). Investigating the direct relationships between various histone modifications and gene expression is important for parsing the regulatory network. The most studied types of histone modification affecting cell states include lysine methylation and acetylation in H3. The same modification in different lysine sites can cause inverse effects on gene expression. H3K4me3 is associated with active transcription, while H3K9me3 and H3K27me3 play an important role in chromatin heterogenization (Bannister and Kouzarides 2011; Clayton *et al.* 1993; Pogo *et al.* 1966). H3K4me3 was mainly distributed at the promoter region while H3K27me3 was enriched at the promoter region and poised enhancer regulated genes that played important roles in the process of differentiation in mouse and human embryonic stem cells (Banaszynski *et al.* 2013; Barski *et al.* 2007; Rada-Iglesias *et al.* 2011). H3K27ac generally localizes in the promoter and enhancer region, and is considered an active marker (Creyghton *et al.* 2010).

Single-cell histone modification sequencing was first reported in 2015, using microfluidics-based chromatin immunoprecipitation and sequencing (ChIP-seq), named Drop-ChIP (Rotem *et al.* 2015). On average 1000 unique reads of H3K4me3 or H3K4me2 were detected per cell. Distinct from previous ChIP-seq methods which fragmented the genome by ultrasonication or micrococcal nuclease, researchers adopted the nuclease or the Tn5 transposase fused to the protein A/G which enabled direct library amplification after antibody enrichment. These ChIP-seq methods include CUT&RUN, CUT&TAG (Kaya-Okur *et al.* 2019; Skene and Henikoff 2017), and were further optimized for single-cell sequencing (Grosselin *et al.* 2019; Wang *et al.* 2019). In these single-cell methods, on average 12,000 unique reads could be obtained in each single cell (Wang *et al.* 2019). Using these methods, it was difficult to unveil the direct regulatory relationship between histone modification and gene expression.

Later, Paired-Tag (parallel analysis of individual cells for RNA expression and DNA from targeted tagmentation by sequencing) was developed by extending from Paired-seq and CUT&TAG to profile one histone modification and the transcriptome in single cells (Kaya-Okur *et al.* 2019; Zhu *et al.* 2019, 2021). This method was applied to reveal the regulatory system, including H3K4me1, H3K4me3, H3K27ac, H3K27me3 and H3K9me3 in the adult mouse frontal cortex and hippocampus. Using combined analysis of histone modifications and the transcriptome, the authors found that many genes may be suppressed by similar mechanisms. For instance, H3K9me3 was mainly enriched in genes related to sensory pathways, especially olfactory receptor genes and vomeronasal receptor genes. H3K27me3 showed distributions on silenced genes relating to embryonic organ development. In addition to gene promoters, histone modifications in the distal *cis*-regulatory elements were also overrepresented. The candidate *cis*-regulatory elements resided in intergenic regions preferentially enriched with H3K9me3, while H3K4me1 and H3K27ac tended to occur in genic regions. Furthermore, a series of *cis*-regulatory element-gene pairs were inferred. An additional high throughput method named Co-TECH was reported recently (Xiong *et al.* 2021). In that work, the researchers integrated single-cell H3K27ac and transcriptome data to explore the regulatory mechanism in hematopoietic stem cell genesis (Xiong *et al.* 2021).

Here we note that simultaneously measuring two or more histone marks at the single-cell level is still unattainable. There are many kinds of histone modifications in a genome, but few of them have been widely investigated, especially in single-cell studies. The antibody-reliant approaches for detecting histone modification slow research progress compared to other, more easily detected epigenomic layers, such as chromatin accessibility and DNA methylation. Moreover, the direct interaction between histone modification and other epigenetic factors remains unstudied, as currently there is no way to detect them simultaneously.

## DNA METHYLOME AND 3D GENOME

3D genome structure refers to the higher-order conformation of chromosomes which brings widely separated functional elements into close spatial proximity to regulate gene expression (Lieberman-Aiden *et al.* 2009; Rowley and Corces 2016). Parsing the folding state of chromosomes can provide deeper insights into the complex relationships between chromatin structure and the functional state of a cell (Rowley and Corces 2016). For instance, topological genome reorganization to express pluripotent stem cell marker genes can reprogram B lymphocytes into pluripotent stem cells (Stadhouders *et al.* 2018). Dynamic 3D chromatin conformation is also highly related to gene expression regulation during neurogenesis and in neuropsychiatric disorders (Lee *et al.* 2019; Rowley and Corces 2016).

The sn-m3C-seq method was initially developed to capture 3D genome structure information and DNA methylation in the same individual cell (Lee *et al.* 2019). Previous studies applied this technique to study neurons in the human cerebral cortex and found there was a chromatin loop surrounding the cell-type-specific marker genes. This included the excitatory neuron marker gene *SATB2*, which exhibited a 1.15 Mb loop between its promoter, and the *LINC01923* locus, and this was only found in excitatory neurons other than interneurons. Together with the DNA methylome dataset, the authors found that DNA methylation was significantly correlated with chromatin 3D structure. For example, the hypomethylation of CTCF binding domains was suitable for CTCF binding and promoted chromatin interactions to form higher-order architecture (Lee *et al.* 2019). Compared to other omics layers, 3D chromatin structure maps play a limited role as indicators for cell-type atlases (Regev *et al.* 2017).

## SPATIAL TRANSCRIPTOMICS

Metazoans contain numerous cell types which spatially interact with each other in complex ways to fulfill physiological roles. However, in most single-cell studies, tissues were dissociated into single-cell or single-nucleus suspensions for amplification and sequencing, resulting in the loss of spatial information (Stahl *et al.* 2016; Wang *et al.* 2009). The spatial architecture of the brain is fundamentally related to its function. The brain’s gross morphology is comprised of different cells in specific locations, and the morphology of each individual neuron, including soma position, dendritic architecture, and axonal projections, determines its roles in functional circuitry (Lein *et al.* 2017). The spatial transcriptome can be used to investigate brain development, neuronal connectivity, and brain disease (Lein *et al.* 2017). On the other hand, the cerebral cortex is a perfect model for the evaluation of spatial transcriptome detection methods, because of its highly organized structure.

To recover spatial information, different strategies have been reported. One of the classical strategies is to transfer cellular mRNA to a solid surface containing spatial barcode primers (Rodriques *et al.* 2019; Stahl *et al.* 2016; Stickels *et al.* 2021; Vickovic *et al.* 2019). According to the barcode information, we can reconstruct gene expression patterns on the tissue surface. Researchers utilized this method to investigate cellular distributions in the mouse olfactory bulb (Stahl *et al.* 2016), which is consistent with the results of high throughput *in situ* hybridization. Incorporating spatial transcriptomics in the mouse spinal cord over the course of amyotrophic lateral sclerosis, Maniatis *et al*. distinguished regional differences for microglia and astrocytes (Maniatis *et al.* 2019). In another study of single-cell spatial transcriptome analysis of the cerebellum (Rodriques *et al.* 2019), the researchers validated the parasagittal bands of gene expression in the Purkinje layer (Barmack *et al.* 2000; Gravel and Hawkes 1990). They confirmed banded expressed genes such as *Aldoc* and *Plcb4* (Bailey *et al.* 2014; Brochu *et al.* 1990; Demilly *et al.* 2011). Cell types defined by transcriptome data were further divided into subpopulations according to their spatial characters (Rodriques *et al.* 2019). Recently, Slide-seq2 was used to analyze the transcriptome of subcellular compartments, where the soma and proximal neuropil of the mouse hippocampus CA1 cells were investigated and more than 200 dendritically enriched genes were identified (Stickels *et al.* 2021).

The above spatial sequencing methods were not precisely to a single-cell resolution. To observe the single-cell resolution of spatial transcriptome information, sci-Space placed the spatially arrayed hashing oligos on a glass slide and released them into cell nuclei in a tissue slide (Srivatsan *et al.* 2021). This method could measure much wider space in a tissue, and the whole E14 mouse embryo was analyzed. The authors found that the distribution of radial glia cells and neurons revealed gradients of developmental maturity in the brain. They also found neuronal migration patterns in different regions consistent with observations of previous studies (Tan *et al.* 2002; Watanabe *et al.* 2018). For the first time, they observed neurons in the midbrain migrating in both radial and tangential directions. In addition, sci-Space was supported as a spatial “scaffold” for the nonspatial single-cell transcriptome atlases since it recovered single-cell transcriptomes with spatial information (Srivatsan *et al.* 2021).

A further strategy for spatial transcriptomics is based on FISH. Single-molecule FISH (smFISH) is capable of detecting single transcripts, but it could only measure one to four genes simultaneously in its first application (Raj *et al.* 2008). By barcoding, the transcripts within individual cells through multiple rounds of hybridizations, sequential FISH (seqFISH) could detect over ten thousand genes in a tissue section (Eng *et al.* 2019; Shah *et al.* 2018). There are also other methods such as multiplexed error-robust fluorescence in situ hybridization (MERFISH) and spatially-resolved transcript amplicon readout mapping (STAR-map) (Moffitt *et al.* 2018; Wang *et al.* 2018). MERFISH used combinational labeling probes to identify each RNA species by multiple distinct signals. Up to one thousand genes in each single cell can be tested by using this method (Chen *et al.* 2015; Moffitt *et al.* 2018; Moffitt and Zhuang 2016). The cellular orchestra of the neural system in a spatial pattern has been directly presented by these techniques. For example, the mouse hypothalamic preoptic region was investigated by MERFISH (Chen *et al.* 2015), the primary visual cortex was mapped by STAR-map (Wang *et al.* 2018) and the subventricular zone and olfactory bulb in the mouse brain was profiled by seqFISH (Eng *et al.* 2019). Compared to the spatial barcode-based technologies, FISH based technologies could achieve subcellular resolution, but the detection ability relies on gene probes.

The spatial transcriptome methods discussed above have shed light on spatial atlases and spatial blueprints. In addition, they also promote research into discovering disease-related factors and crosstalk of different cell types in a microenvironment (Liao *et al.* 2021). Since many neural diseases were highly correlated with brain regions, these techniques should play an increasingly important role in future research.

## TRANSCRIPTOME AND PROTEOME

As the terminal executors, proteins determine cell structure, metabolic reactions, and cell function (Labib and Kelley 2020). Direct proteome detection offers clues on the cellular responses to genomic, epigenomic, and environmental alterations. Investigating the proteome can help us to determine key functional markers and understand pathogenicity mechanisms in different diseases (Aslam *et al.* 2017). Further integrating the proteome with other omics layers such as the epigenome and transcriptome shows great potential in revealing novel insights into biological regulation (Zhang and Kuster 2019). Different from genome and transcriptome sequencing, which rely on the arrangement and combination of the four nucleotides A, T, C, G, proteins consist of 20 amino acids which are not detectable by the sequencers. The currently achievable way to resolve the proteome is partially by mass spectrometry. The essential chemical difference between the DNA/RNA sequence and the amino acid sequence sets a barrier to sequencing the proteome with other omics layers.

In recent years, researchers measured the abundance of specific proteins by transforming them to antibody-coupled DNA/RNA fragments. This way, these proteins could be detected simultaneously with the transcriptome within the same cell. Such techniques include PEA/STA (proximity extension assays/specific (RNA) target amplification), REAP-seq (RNA expression and protein sequencing assay), SPARC (single-cell protein and RNA co-profiling) and CITE-seq (cellular indexing of transcriptomes and epitopes by sequencing) (Genshaft *et al.* 2016; Peterson *et al.* 2017; Reimegard *et al.* 2021; Stoeckius *et al.* 2017). PEA/STA, REAP-seq and CITE-seq are only available for cell surface proteins while SPARC was developed for investigating the intercellular proteome. Each of those methods relied on the efficiencies of antibodies. This way, they could only detect limited protein types with accessible antibodies, while other proteins could not be studied by this method. Also, protein modifications including glycosylation, neddylation, SUMOylation and others can’t be distinguished through these methods.

Mass cytometry can also be used to detect proteins, such as PLAYR (proximity ligation assay for RNA), which can simultaneously detect more than 40 different protein epitopes and RNA targets in thousands of cells per second (Frei *et al.* 2016). PLAYR has the ability to detect both cell-surface and cytoplasmic proteins. But with a limited number of available tags, the proteins and RNA that can be detected in individual cells are also quite limited.

## FUTURE PERSPECTIVES

The rapid development of single-cell multi-omics sequencing technology has enabled more accurate research into a series of biological processes, including disease and development. Fittingly, this technique was announced as ‘Method of the Year 2019’ by Nature Methods (https://doi.org/10.1038/s41592-019-0703-5). Even so, we must note the tradeoff between breadth and sensitivity in including expanded sets of omics data. That is, compared to single-cell mono-omics sequencing, multi-omics sequencing harvests more sparse data. SNARE-seq and Paired-seq detected less than one quarter of DNA reads compared to single-cell combinatorial indexing protocols for chromatin accessibility (sci-ATAC-seq) (Chen *et al.* 2019; Cusanovich *et al.* 2018; Zhu *et al.* 2019). The triple-omics method snmC2T detected fewer useful reads than the dual-omics method snmCT (Luo *et al.* 2019). On the other hand, different omics layers each have their own advantages, for example, the DNA methylome outperformed the 3D genome in distinguishing cell types (Lee *et al.* 2019), and chromatin accessibility could better elucidate the regulations of cell lineage specifications (Ma *et al.* 2020b). Therefore, it is necessary to weigh those advantages and disadvantages before selecting an optimal research method for a given question.

Single-cell analysis, especially single-cell multi-omics analysis, is vital to understanding systems of neural circuits as they comprise enormous numbers of neural cell types with complex regulation networks and experience-dependent plasticity (Armand *et al.* 2021). The development of technology has remarkably accelerated understanding of the cellular diversity of the nervous system and provided insights into the molecular regulatory networks regarding neural development and diseases. However, except for the integrated single-cell analysis of the transcriptome and chromatin accessibility or DNA methylation (Chen *et al.* 2019; Luo *et al.* 2019; Zhu *et al.* 2019), other multi-omics combinations have not shed light on their key roles in the neural system so far. For example, histone modifications and 3D genome structures are also essential for the nervous system (Hon *et al.* 2013; Li *et al.* 2018; Stadhouders *et al.* 2018, 2019), yet we still do not know how these epigenetic factors participate in neural fate commitment. Moreover, the key pathogenic mechanisms underlying neuropsychiatric diseases such as ASD and Alzheimer’s Disease still need further investigation (Breijyeh and Karaman 2020). Specific to neurons, omics information must also correspond to morphologies and electrophysiological characteristics under stimulation, thus defining the molecular determinants of neuronal function. The evolutionary relationships among neural populations within the same species and across species still need further investigation. In summary, there are still many open questions regarding the nervous system, many of which are best addressed by evolving technologies of single-cell multi-omics sequencing.

## Conflict of interest

Chaoyang Wang and Xiaoying Fan declare that they have no conflict of interest.
